# Effects of motor–cognitive training on dual-task performance in people with Parkinson’s disease: a systematic review and meta-analysis

**DOI:** 10.1007/s00415-023-11610-8

**Published:** 2023-02-23

**Authors:** Hanna Johansson, Ann-Kristin Folkerts, Ida Hammarström, Elke Kalbe, Breiffni Leavy

**Affiliations:** 1 Division of Physiotherapy, Department of Neurobiology, Care Sciences and Society, Karolinska Institutet, Alfred Nobels Allé 23, Huddinge, 14183 Stockholm, Sweden; 2 Karolinska University Hospital, Theme Womens Health and Allied Health Professionals, Stockholm, Sweden; 3 Medical Psychology | Neuropsychology and Gender Studies, Centre for Neuropsychological Diagnostics and Intervention (CeNDI), Faculty of Medicine and University Hospital Cologne, University of Cologne, Cologne, Germany; 4 Stockholm Sjukhem Foundation, Mariebergsgatan 22, 112 19 Stockholm, Sweden

**Keywords:** Systematic review, Meta-analysis, Parkinson’s disease, Motor–cognitive training, Dual-task performance

## Abstract

Motor–cognitive training in Parkinson’s disease (PD) can positively affect gait and balance, but whether motor–cognitive (dual-task) performance improves is unknown. This meta-analysis, therefore, aimed to establish the current evidence on the effects of motor–cognitive training on dual-task performance in PD. Systematic searches were conducted in five databases and 11 studies with a total of 597 people (mean age: 68.9 years; mean PD duration: 6.8 years) were included. We found a mean difference in dual-task gait speed (0.12 m/s (95% CI 0.08, 0.17)), dual-task cadence (2.91 steps/min (95% CI 0.08, 5.73)), dual-task stride length (10.12 cm (95% CI 4.86, 15.38)) and dual-task cost on gait speed (− 8.75% (95% CI − 14.57, − 2.92)) in favor of motor–cognitive training compared to controls. The GRADE analysis revealed that the findings were based on high certainty evidence. Thus, we can for the first time systematically show that people with PD can improve their dual-task ability through motor–cognitive training.

**Supplementary Information:**

The online version contains supplementary material available at 10.1007/s00415-023-11610-8.

## Background

Everyday life requires us to perform tasks simultaneously and pay attention while doing so. This act of dual-tasking is by definition conducted when two tasks with distinct goals are performed simultaneously [1]. People with neurological disorders typically experience greater difficulties while dual-tasking compared to healthy controls [2, 3]. In Parkinson’s disease (PD) specifically, gait impairments are well documented and show that during dual-task walking conditions, gait speed [4–14] and step length [4–14] decrease, while gait variability [4, 8, 9, 12–14], and the number of freezing episodes increase [15]. Interview studies with people with PD elucidate the need to concentrate to maintain a basic walking rhythm even at early stages of the disease, [16] and to use self-talk to anticipate and plan for the next step ahead [17].

Although not yet fully understood, it is believed that executive dysfunction, together with a gradual loss of automaticity in PD may partly explain the impaired ability to dual task [18]. According to the model proposed by Fitts and Posner (1967), motor learning occurs through a three-stage process [19]. At first, we familiarize ourselves with the task through conscious performance and information processing (cognitive stage). In the second, associative stage, we start carrying out the task, adjust it and finetune its performance. Finally, in the autonomous stage, the task can be performed with minimal cognitive and attentional demand [19]. As a movement becomes automatic, imaging studies on the healthy brain have shown that brain activity in the dorsolateral prefrontal cortex and the anterior cingulate cortex decreases, whereas connectivity increases between the putamen and different motor areas. However, in PD, due to dopamine depletion in the putamen, no such connectivity increase occurs, resulting in difficulties acquiring automaticity [18, 20].

The extent to which people with PD can improve their ability to dual task through training is unclear, despite an increase in the number of trials focusing on motor–cognitive training during the past decade. This is because previous efforts to systematically study the effects of motor–cognitive training in PD [21–23] have focused on effects on single-task gait and balance, and not on dual-task performance per se. In consideration of the principles of motor learning, combining motor and cognitive training (motor–cognitive training) should provide the added advantage of task specificity, when compared to the consecutive training of these tasks. However, it has also been debated whether in certain PD subgroups, such as individuals with cognitive impairment or those suffering from freezing of gait, consecutive training is a safer option [24]. The prevalence of both these symptoms is reported as 40%, even at early disease stages [25, 26], and increases in line with disease progression [26, 27].

In older adults at various stages of cognitive impairment, motor–cognitive training has shown beneficial effects for both physical [28, 29] and cognitive function, as well as in reducing dual-task cost on gait speed (i.e., the proportion by which gait speed is reduced compared to single-task walking) [28]. Interestingly, motor–cognitive training also appears to have a larger effect on executive function than cognitive training alone which suggests that physical exercise might act as an aggregate [30]. Nonetheless, due to the lack of systematic evidence to support whether dual-task training techniques can improve motor–cognitive function in PD, it is unknown whether these more complex interventions lead to benefits for this group when performing dual-task activities. Thus, the aim of this systematic review and meta-analysis is to establish the current evidence on the effects of motor–cognitive training on dual-task performance in people with PD.

## Methods

This systematic review adhered to the Preferred Reporting Items for Systematic Reviews and Meta-analysis (PRISMA) statement [31]. It was preregistered in the International Prospective Register of Systematic Reviews (PROSPERO; CRD42021278518).

### Keywords, databases, and review process

Searches were conducted by information specialists in the following databases up to September 28th, 2021, CINAHL, Web of Science, MEDLINE Ovid and Cochrane Central Register of Controlled Trials (CENTRAL). Reference lists of all studies that were found to be eligible for this review were hand searched for further eligible trials. Language was restricted to English, Swedish, and German. No restrictions were set for publication date. Databases were searched with an elaborate search string (see Online Resource 1).

Studies retrieved through the electronic database searches were screened based on title and abstract independently by two review authors (HJ and IH) in Rayyan [32]. After completed screening, authors were unblinded to each-others’ decisions. Disagreements were discussed and resolved with other members (BL, EK and AKF) of the review team. After the initial screening, a full text review of the included studies was performed independently by two authors (HJ and IH), and unblinded upon completion. In case of uncertainty, further review authors (BL, EK and AKF) were consulted until consensus was reached. Final decisions for inclusion were hereafter discussed within the review team.

### Eligibility criteria

Eligible study designs were randomized controlled trials (RCT) or quasi-RCT. Reviews and meta-analysis were excluded as well as letters to the editors, comments, conference posters, and further conference contributions, study protocols and trial register entries, books and book chapters were excluded. Studies were eligible for inclusion if they were conducted on human adults (≥ 18 years) of all sexes with a clinical diagnosis of idiopathic PD. Data from patients with atypical, genetic, or secondary Parkinsonism were not included. Regarding interventions, we focused on motor–cognitive training (dual-task training), i.e., training involving motor tasks (e.g., walking) and cognitive tasks (e.g., counting down) performed simultaneously. Motor–cognitive interventions were eligible regardless of approach (e.g., traditional or virtual reality/exergaming) and setting (in-clinic or home-based), but the minimum number of training sessions needed to be ≥ 2. Both passive (no exercise or other type of organized activity) and active (e.g., exercise without elements of dual-tasking, or education) control groups were eligible. Reporting of any type of measurable dual-task performance (e.g., dual-task gait speed) was required for inclusion.

### Data extraction

A pre-piloted form was used to extract data from the included studies. Extracted information included: participant demographics, details of disease stage and cognitive function, details of the motor–cognitive intervention and the control intervention; details of the dual-task outcomes pre and post intervention pertaining to both motor task and cognitive task.

One review author (IH) extracted the data, and another reviewer (HJ) double-checked it. Ambiguity was resolved through discussion where necessary. Missing data was requested from study authors. If data were still not obtained after two reminders to study authors, the data were considered as missing. Data were requested from six reports [33–38], whereof data from four reports were retrieved [33, 36–38].

### Data analyses

Three studies had more than one report included, and for this reason only the main reports [36, 38, 40] were referenced for sample characteristics, and more than one report for each study never included in the same meta-analysis. One study [36] was a cross-over RCT, and therefore only midpoint data was used in the meta-analyses. Descriptive data on study participants were pooled among the studies (i.e., not reports).

Review Manager (RevMan) version 5.4 was used for meta-analyses [41]. The outcomes available for meta-analyses (dual-task gait speed, dual-task cadence, dual-task stride length, dual-task stride length standard deviation (SD), dual-task stride time SD, dual-task double support, dual-task cost on gait speed, dual-task reaction time, Timed Up and Go cognitive (TUG cog)) were continuous and treatment effect measures were therefore given as mean differences (MDs) with 95% confidence intervals (CI). As we expected some heterogeneity in the trial designs, a random-effect model was used. Additional outcomes that were only reported by a single study (i.e., dual-task cost on stride length, dual-task accuracy, dual-task cost on accuracy, and dual-task unipedal stance test) were also meta-analyzed and can be found in Online Resource 2, Fig. 1a–d. Two types of sensitivity analyses were performed; one comparing meta-analyses using fixed-effect models, and one comparing meta-analyses with and without including studies using a passive control group [38].

### Risk of bias assessment

Risk of bias was assessed on outcome level (dual-task performance) using Cochrane Risk of Bias tool 2.0 (RoB2) which considers bias arising from the randomization process, bias due to deviations from intended interventions, bias due to missing outcome data, bias in measurement of the outcome, bias in selection of the reported result and overall risk of bias. Two authors (HJ and BL) assessed risk of bias using RoB2 independently and unblinded after completion, whereafter any discrepancies were resolved through discussion with a third author (AKF).

In accordance with our protocol registration, no funnel plots for publication bias were created as each of the meta-analyses performed included less than ten studies according to the Cochrane Handbook for Systematic Reviews of Interventions [42]. Publication bias was instead assessed by searching trial registries (ClinicalTrials.gov and International Clinical Trials Registry Platform (ICTRP)) to identify completed but not published trials. In cases where no publication could be retrieved from a completed trial, the principal investigator of the respective trials was contacted in order to obtain more information. Principal investigators of the following trial register entries were contacted: NCT03902990, RBR-365tkt, NCT01156714, and NCT02904837, without response.

Two authors (HJ and BL) independently assessed the certainty of the body of evidence for studies that contributed to the meta-analyses using the five GRADE considerations (study limitations, consistency of effect, imprecision, indirectness, and publication bias). The GRADEpro GDT software was used to prepare the Summary of findings tables (GRADEpro GDT 2022) [43]. Any decisions to downgrade the certainty of studies were justified in footnotes.

## Results

### Study selection

**Fig. 1:**
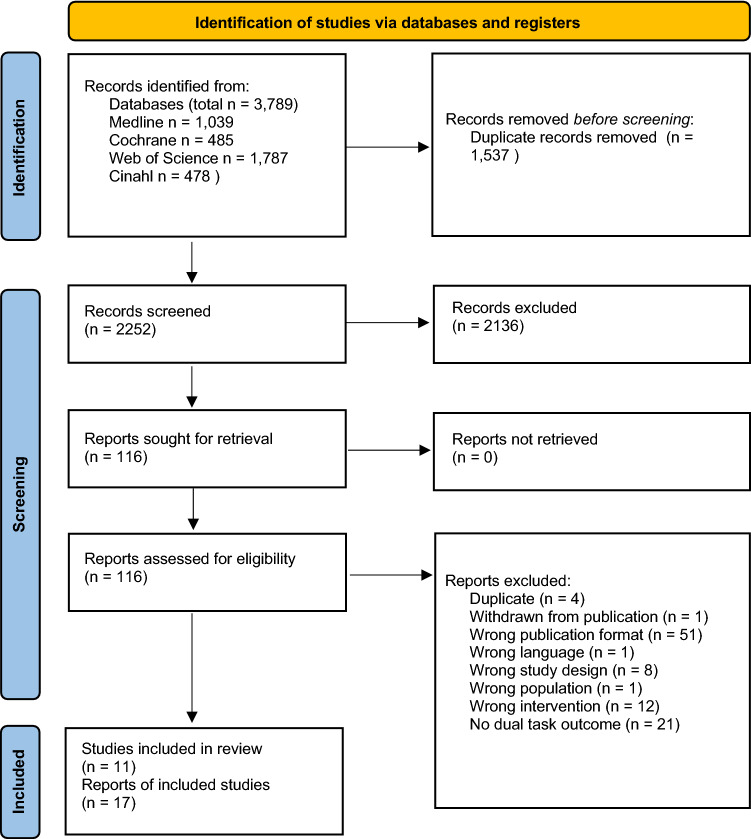
PRISMA flow diagram of the screening process. Modified from Page et al. [44]

The database searches up to September 2021 yielded a total of 2252 records after duplicates were removed. A total of 2136 records were excluded based on the aforementioned eligibility criteria, leaving 116 records for full text evaluation. During full text evaluation, 99 reports were excluded, see Online Resource 3, Table 1, for detailed information on reasons for exclusion. A final of 11 studies (reported in 17 articles) were included for further qualitative and quantitative analyses. See Fig. 1 for a PRISMA flow diagram of the screening process.

### Study characteristics

#### Population characteristics

All included studies were of RCT design and had been conducted in the following countries: Belgium, Israel, the Netherlands, Spain, Sweden, Taiwan, and the United States of America (USA). A total of 597 people were randomized into the studies, for which descriptive characteristics were reported in 573 (37.2% women) and 564 were analyzed post the interventions. Participants included in the studies had a pooled mean age of 68.9 years (pooled SD 7.4), a pooled mean H&Y of 2.0 (pooled SD 0.4), and a pooled mean disease duration of 6.8 years (pooled SD 4.8). Six reports [35–37, 40, 45, 46] from two main studies [36, 40] stated the percentage of individuals suffering from freezing of gait (FOG) in their respective samples. Five reports [35, 36, 46–48] described FOG symptoms using either the freezing of gait questionnaire (FOGQ) [47, 48] or the new FOGQ (NFOGQ) [35, 36, 46]. Two studies used severe motor fluctuations as an exclusion criterion [47, 48], but none of the included studies described the occurrence or severity of motor fluctuations in their respective samples. Levodopa equivalent daily dosage (LEDD) was described in 11 [33, 38–40, 45–51] of the 17 reports. Nine studies used a cutoff for global cognition as an inclusion criteria [33, 34, 38, 40, 48, 49, 51–53], one study [36] used ability to consent and follow testing and intervention procedures as an inclusion criteria, and one study [47] did not specify any exclusion based on cognitive ability. For information on individual reports and characteristics on included participants, see Table 1.

**Table 1:** Description of reports and characteristics of included samples

References	Country	Type of analysis	Group	N randomized	Percent female	Age (years), mean (SD)	Disease duration (years), mean (SD)	LEDD (mg), mean (SD)	Occurrence of freezing, % Freezers, and/or FOGQ or NFOGQ, mean (SD)	H&Y, mean (SD)	Cognitive function, Test: mean (SD)
Conradsson et al. [38] (2015)	Sweden	Per protocol	Intervention	51	40.4	72.9 (6.0)	6.0 (5.1)	581 (295)	NI	2.6 (0.5)	NI
			Control	49	48.9	73.6 (5.3)	5.6 (5.0)	645 (404)	NI	2.6 (0.5)	NI
Geroin et al. (2018) [45]	Belgium/The Netherlands	Intention to treat	Intervention	56	30.4	65.8 (9.19)	8.4 (5.3)	613 (396)	60.7	2.3 (0.5)	MMSE: 28.0 (1.5)
			Control	65	24.6	66.05 (9.3)	8.9 (5.3)	752 (453)	52.3	2.3 (0.5)	MMSE: 27.9 (1.7)
Hasegawa et al. [37] (2020)	United States	Per protocol	Intervention	47	31.8	67.7 (6.7)	6.2 (4.4)	NI	52.3	2.1 (0.4)	MoCA: 26.5 (2.9)
			Control	46	33.3	70.0 (8.2)	6.7 (5.5)	NI	45.2	2.4 (0.8)	MoCA: 24.6 (3.9)
Johansson et al. [49] (2020)	Sweden	Per protocol	Intervention	7	14.3	70.9 (6.2)	9.0 (3.5)	*700 (233)	NI	2.3 (0.5)	MoCA: 27.3 (1.5)
			Control	6	50.0	67.3 (2.8)	6.8 (2.7)	*766 (322)	NI	2.5 (0.5)	MoCA: 27.7 (2.5)
Jung et al. [36] (2020)	United States	Per protocol	Intervention	47	31.8	67.7 (6.7)	6.2 (4.4)	NI	52.3 NFOGQ: 6.7 (7.9)	2.1 (0.4)	SCOPA-cog: 28.8 (4.8)
			Control	46	33.3	70.0 (8.2)	6.7 (5.5)	NI	42.5 NFOGQ: 5.2 (7.4)	2.4 (0.8)	SCOPA-cog: 27.5 (4.9)
King et al. [35] (2020)	United States	Per protocol	Intervention	25	NI	68.2 (5.2)	7.3 (4.3)	NI	100	2.4 (0.7)	MoCA: 26.6 (3.0)
			Control	21	NI	69.1 (9.5)	9.7 (6.0)	NI	100	2.6 (0.9)	MoCA: 24.3 (4.2)
Lofgren et al. [50, 54] (2019)	Sweden	Per protocol	Intervention	51	40.0	72.5 (5.8)	5.8 (4.7)	592 (287)	NI	2.6 (0.5)	NI
			Control	49	50.0	73.5 (5.6)	5.4 (4.7)	640 (422)	NI	2.6 (0.5)	NI
Maidan et al. [33] (2018)	Israel	Intention to treat	Intervention	30	26.7	70.1 (7.1)	8.9 (6.0)	833 (102)	NI	NI	MMSE: 28.2 (1.6)
			Control	34	32.4	73.1 (6.4)	9.7 (5.8)	1186 (238)	NI	NI	MMSE: 28.3 (1.8)
Pohl et al. [47] (2020)	Sweden	Per protocol	Intervention	28	26.9	69.7 (7.0)	6.0 (4.4)	728 (327)	FOGQ: 6.0 (4.3)	2.4 (0.7)	MoCA: 25.5 (2.8)
			Control	23	35.0	70.4 (6.0)	6.8 (3.6)	690 (231)	FOGQ: 5.2 (4.3)	2.3 (0.7)	MoCA: 25.0 (3.3)
Pompeu et al. [34] (2012)	Brazil	Intention to treat	Intervention	16	NI	68.6 (8.0)	4.7 (5.4)	NI	NI	NI	MMSE: 26.4 (2.2)
			Control	16	NI	66.2 (8.3)	5.2 (3.4)	NI	NI	NI	MMSE: 27.3 (2.6)
Rosenfeldt et al. [51] (2019)	United States	Per protocol	Intervention	10	10	59.0 (9.0)	8 (4.12)*	***437.5 (405–916.3)	NI	2.4 (0.5)	NI
			Control	11	50	65.0 (8.0)	4 (3.6)*	***550 (412.5–756.5)	NI	2.2 (0.4)	NI
San Martín Valenzuela et al. [52] (2020)	Spain	Per protocol	Intervention	24	52.2	66.4 (7.1)	6.3 (6.0)	NI	NI	2.6 (0.6)	NI
			Control	23	29.4	64.8 (8.8)	5.3 (3.8)	NI	NI	2.5 (0.7)	NI
Strouwen et al. [40] (2017)	Belgium/The Netherlands	Intention to treat	Intervention	56	30.4	65.8 (9.19)	8.4 (5.3)	613 (396)	60.7	2.3 (0.5)	MMSE: 28.0 (1.5)
			Control	65	24.6	66.05 (9.3)	8.9 (5.3)	752 (453)	52.3	2.3 (0.5)	MMSE: 27.9 (1.7)
Strouwen et al. [46] (2019)	Belgium/The Netherlands	Intention to treat	Intervention	56	30.4	65.8 (9.19)	8.4 (5.3)	613 (396)	60.7 NFOGQ: 2.0 (0.0–14.0)*	2.3 (0.5)	MMSE: 28.0 (1.5)
			Control	65	24.6	66.05 (9.3)	8.9 (5.3)	*710 (427.50–931.67)	52.3 NFOGQ: 0.0 (0.0–13.0)*	2.3 (0.5)	MMSE; 27.9 (1.7)
Wallen et al. [39] (2018)	Sweden	Intention to treat	Intervention	51	37.3	73.1 (5.8)	5.9 (5.1)	578 (299)	NI	2.5 (0.5)	MMSE: 28.0 (1.5)
			Control	49	49.0	73.0 (5.5)	5.6 (4.8)	640 (380)	NI	2.6 (0.5)	MMSE: 28.0 (1.8)
Yang et al. [48] (2019)	Taiwan	Intention to treat	Intervention	6	33.3	66.7 (10.9)	6.7 (3.7)	**892 (432–1308)	FOGQ: 8.5 (2.3–11.7)**	2.1 (0.5)	MMSE: 27.0 (25.9–28.1)**
			Control 1^a^	6	33.3	71.0 (6.5)	6.3 (5.9)	**798 (535–1074)	FOGQ: 13.0 (2.3–19.4)**	2.3 (0.4)	MMSE: 27.5 (25.1–28.9)**
			Control 2^b^	6	33.3	66.0 (12.5)	5.2 (4.6)	**557 (208–1235)	FOGQ: 4.5 (0.0–9.3)**	1.7 (0.7)	MMSE: 28.0 (27.1–28.9)**
Yen et al. [53] (2011)	Taiwan	Intention to treat	Intervention	14	85.7	70.4 (6.5)	6.0 (2.9)	NI	NI	2.6 (0.5)	MMSE: 28.5 (1.6)
			Control^c^	14	85.7	70.1 (6.9)	6.1 (3.3)	NI	NI	2.4 (0.5)	MMSE: 28.5 (1.2)
			Control 2^d^	14	64.3	71.6 (5.8)	7.8 (4.2)	NI	NI	2.6 (0.4)	MMSE: 28.1 (0.8)

*FOGQ* Freezing of Gait Questionnaire, *H&Y* Hoehn and Yahr, *LEDD* Levodopa Equivalent Daily Dosage, *MMSE* Mini Mental State Examination, *MoCA* Montreal Cognitive Assessment, *NFOGQ* New Freezing of Gait Questionnaire, *NI* no information, *SCOPA-cog* Scales for Outcomes in Parkinson's Disease-COGnition

*Median (IQR); **Median (95% CI); ***Median (25th percentile, 75th percentile)

^a^Motor dual-task training

^b^General gait training

^c^Conventional balance training

^d^No training

#### Interventions and comparisons

The content of the motor–cognitive interventions varied and included highly challenging dual-task balance training [38, 49], circuit training progressed with cognitive dual tasks [36], treadmill training with virtual reality (VR) [33], Wii-based motor and cognitive training [34], dual-task gait training [40, 48, 52], and balance training with VR [53]. The dose ranged between 30 and 80 min (mean 51.8), 2–4 times per week (mean 2.6) for 4–12 weeks (mean 7.7). Two studies used an added home training program to the motor–cognitive intervention to be performed for an additional 60 min per week [40, 49]. Six of the interventions were conducted in a group setting [36, 38, 49, 52], and five as individual training [33, 34, 40, 48, 53]. Two studies used a passive control group [38, 47], one study used both a passive and an active control group [53], seven studies used an active control group [33, 34, 36, 40, 49, 52], and one study used two different active control groups [48]. Active control group content included gait and cognitive training performed consecutively [40, 51], gait training [52], motor dual-task gait training [48], balance training [53], treadmill training [33], global exercises and balance training [34], speech and communication therapy [49], and education [36]. The dose of the active control group interventions ranged between 30 and 80 min (mean 48.3), 1–4 times per week (mean 2.4) for 4–10 weeks. Three studies used an added home training program in addition to the active control group training, and these increased the weekly training by 60–180 min. For detailed information on the content of each intervention, see Table 2.

**Table 2:** Description of interventions in included studies (associated reports listed below)

References	Motor–cognitive intervention		Control intervention	
	Description	Dose	Description	Dose
Conradsson et al. [38] (2015)^a^	Highly challenging balance-training incorporating dual-tasking and PD-specific balance components. Performed in groups of 4–7 participants	60 min, 3 times/week for 10 weeks	Care as usual	NA
Johansson et al. [49] (2020)	Highly challenging balance-training incorporating dual-tasking and PD-specific balance components. Performed in groups of 4–7 participants + HEP (aerobic capacity (for example walking), leg and core strength exercises)	60 min, 2 times/week for 10 weeks HEP: 60 min, once a week for 10 weeks	Speech and communication therapy. Performed in groups of 4–7 participants + HEP (relaxation & breathing, voice & speech, and word & memory exercises)	60 min, 2 times/week for 10 weeks HEP: 60 min, once a week for 10 weeks
Jung et al. [36] (2020)^b^	Circuit training involving (1) gait, (2) PWR! Moves©, (3) agility, (4) lunges, (5) boxing, and (6) adapted Tai Chi. Progression at three levels of difficulty by varying visual and surface condition (and other), and through adding cognitive secondary tasks	80 min, 3 times/week for 6 weeks	Education program focused on self-management of care team development, nutrition, mood, sleep, stress and medication + HEP (relaxation CD’s)	80 min, 1 time/week for 6 weeks HEP: 30 min, 6 times/week for 6 weeks
Maidan et al. [33] (2018)	Treadmill training with virtual reality for an obstacle navigation task. Individual training	45 min, 3 times/week for 6 weeks	Treadmill training. Individual training	45 min, 3 times/week for 6 weeks
Pohl et al. [47] (2020)	Exercises typical for the Ronnie Gardiner Method (initiated with soft stretching and breathing exercises, followed by 50 min of exercises according to the Ronnie Gardiner Method, and ended with winding down). Performed in two groups of 12 and 14 participants respectively	60 min, 2 times/week for 12 weeks	Care as usual	NA
Pompeu et al. [34], (2012)	Global exercises (10 min warm-up, 10 min of resistance exercises, and 10 min of exercises in diagonal patterns). Wii-based motor and cognitive training for 30 min. Individual training	60 min (30 min global and 30 min Wii), 2 times/week for 7 weeks	Global exercises (10 min warm-up, 10 min of resistance exercises, and 10 min of exercises in diagonal patterns). Balance training mimicking the same movements and time required by each game in each trial, for 30 min. Individual training	60 min (30 min global and 30 min balance), 2 times/week for 7 weeks
Rosenfeldt et al. [51] (2019)	Gait training and cognitive training performed simultaneously. During gait training, high velocity, high amplitude training principles with an external focus of attention was used. Cognitive training involved attention, memory, language, and executive function	45 min, 3 times/week for 8 weeks	Gait training followed by cognitive training, each performed separately. Same training principles as explained in motor–cognitive intervention group	45 min, 3 times/week for 8 weeks
San Martín Valenzuela et al. [52] (2020)	Dual-task program: 10 min warm-up, 45 min of dual-task gait training, and 5 min of cool-down. Performed in groups of max 10 participants	60 min, 2 times/week for 10 weeks	Single task program: 10 min warm-up, 45 min of single-task gait training, and 5 min of cool-down. Performed in groups of max 10 participants	60 min, 2 times/week for 10 weeks
Strouwen et al. [40] (2017)^c^	Integrated task training; 30 min of concurrent practice of gait and cognitive exercises and 10 min of functional training with integrated dual-task practice. Individual training performed in the home + HEP	40 min, 2 times/week for 6 weeks HEP: 30 min, 2 times/week for 6 weeks	Consecutive task training; 15 min of gait training, 15 min of cognitive exercises and 10 min of functional practice. Individual training performed in the home + HEP	40 min, 2 times/week for 6 weeks HEP: 30 min, 2 times/week for 6 weeks
Yang et al. [48] (2019)	Cognitive dual-task gait training	30 min, 3 times/week for 4 weeks	Control 1: Motor dual-task gait training (MDTT) Control 2: General gait training	30 min, 3 times/week for 4 weeks
Yen et al. [53] (2011)	Virtual reality augmented balance training	30 min, 2 times/week for 6 weeks	Control 1: Conventional balance training Control 2: No training	Control 1: 30 min, 2 times/week for 6 weeks Control 2: NA

*HEP* home exercise program, *NA* not applicable

Associated reports:

^a^Lofgren et al. [50] and Wallen et al.[39]

^b^King et al. [35] and Hasegawa et al.[37]

^c^Geroin et al. [45] and Strouwen et al. [46]

#### Outcomes of dual-task performance

Various spatiotemporal aspects of gait during dual-task walking were the most commonly reported outcomes of dual-task performance in the included studies. Most frequent was the reporting of dual-task gait speed, followed by cadence, stride (or step) length, percent of the gait cycle spent in double support, and stride length- and stride time variability (standard deviations). Five studies reported dual-task performance of a cognitive task (e.g., reciting every other letter of the alphabet, an auditory Stroop task, and a subtraction task) that was carried out simultaneously with a motor task, and presented in terms of accuracy rate, dual-task cost on accuracy rate, error rate and/or reaction time [36, 38, 40, 49, 53]. Other assessments evaluating dual-task performance included the Timed Up and Go cognitive (TUG cog), a dual-task test of functional mobility, and the unipedal stance test performed with a simultaneous cognitive task.

### Results of syntheses

#### Dual-task gait speed

**Fig. 2:**
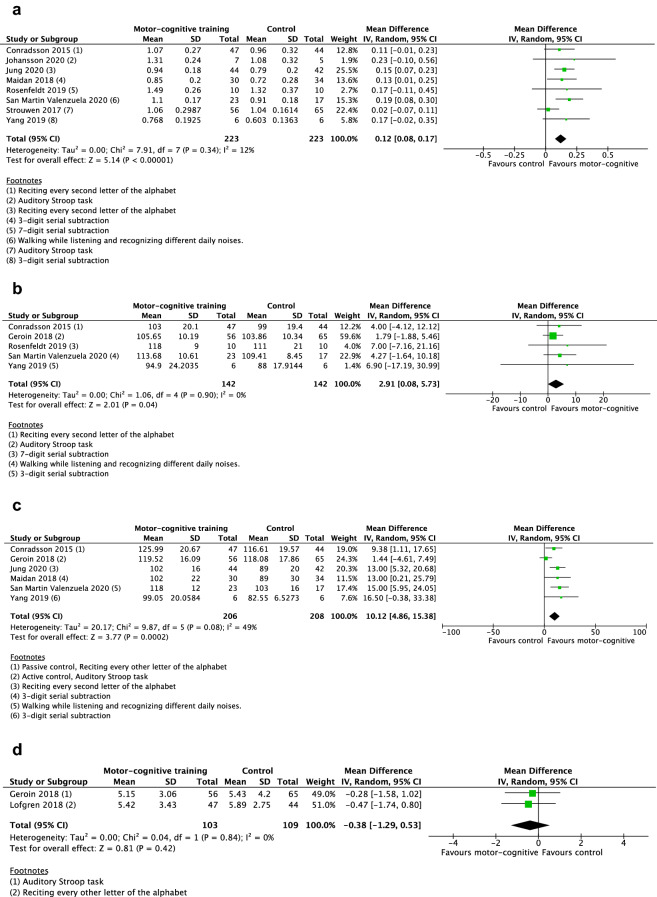

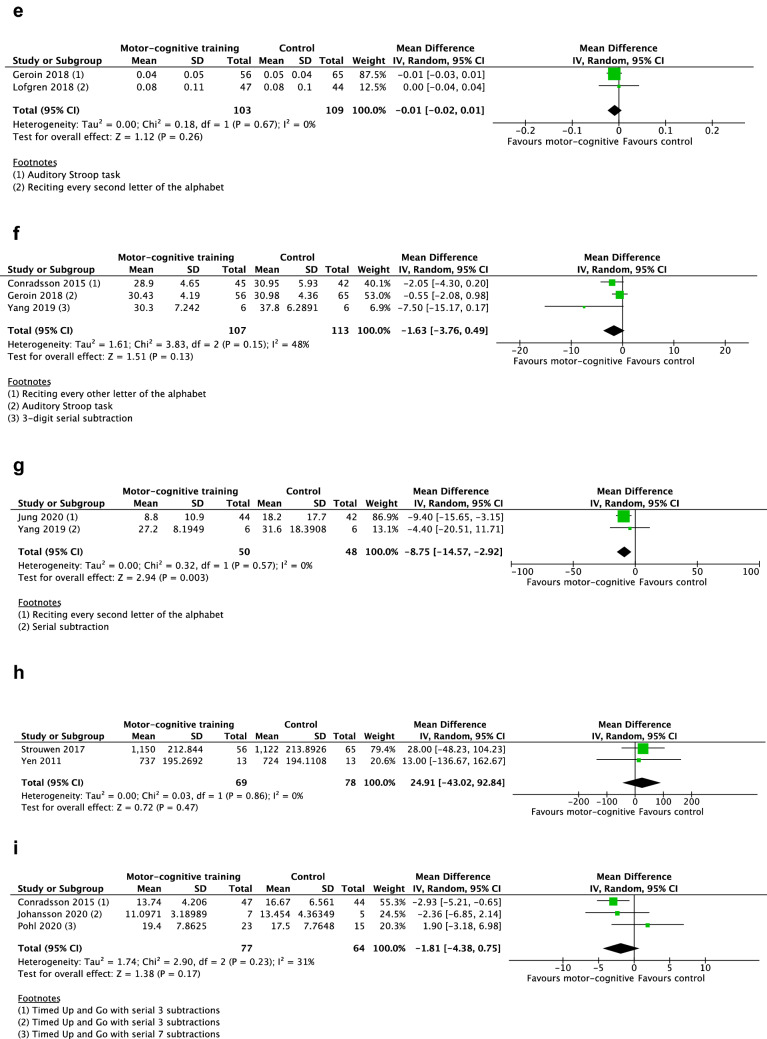
Forest plot. **a** Motor–cognitive training vs control. Outcome: dual-task gait speed. **b** Motor–cognitive training vs control. Outcome: dual-task cadence. **c** Motor–cognitive training vs control. Outcome: dual-task stride length. **d** Motor–cognitive training vs control. Outcome: dual-task stride length SD. **e** Motor–cognitive training vs control. Outcome: dual-task stride time SD. **f** Motor–cognitive training vs control. Outcome: dual-task double support. **g** Motor–cognitive training vs control. Outcome: dual-task cost on gait speed. **h** Motor–cognitive training vs control. Outcome: dual-task reaction time. **i** Motor–cognitive training vs control. Outcome: Timed Up and Go cognitive

Eight studies were included in the meta-analysis for the outcome dual-task gait speed [33, 36, 38, 40, 48, 49, 51, 52], see Fig. 2a. In terms of overall risk of bias, two studies were assessed as having high risk of bias, six studies some concerns, and one study was considered to have low risk of bias. The random-effects model showed a significant mean difference in gait speed of 0.12 m/s (95% CI 0.08, 0.17) in favor of the motor–cognitive training in contrast to passive and active control groups.

#### Dual-task cadence

Five studies were included in the meta-analysis for the outcome dual-task cadence [38, 45, 48, 51, 52], see Fig. 2b. Two studies had high overall risk of bias, two studies were assessed as some concerns, and one study as low. The random-effect model showed a significant mean difference cadence of 2.91 steps/min (95% CI 0.08, 5.73) in favor of the motor–cognitive training in contrast to passive and active control groups.

#### Dual-task stride length

Six studies were included in the meta-analysis for the outcome stride length [33, 36, 38, 45, 48, 52], see Fig. 2c. Two studies had high overall risk of bias, three studies were assessed as some concerns, and one study as low. The random-effect model showed a significant mean difference in stride length of 10.12 cm (95% CI 4.86, 15.38) in favor of the motor–cognitive training in contrast to passive and active control groups.

#### Dual-task gait variability

Two studies reported synthesizable data on gait variability [45, 50]. One study had low overall risk of bias, and one had some concerns. Neither of the two random-effect models showed significant results regarding stride length SD (*p* = 0.42), Fig. 2d, or stride time SD (*p* = 0.26), Fig. 2e.

#### Dual-task double support

Three studies were included in the meta-analysis for the outcome double support [38, 45, 48], see Fig. 2f. One study had low overall risk of bias, one had some concerns, and one had high. The random-effects model was not significant (*p* = 0.13) regarding any mean difference in double support between motor–cognitive training and control.

#### Dual-task cost on gait speed

Two studies were included in the meta-analysis for the outcome dual-task cost on gait speed [36, 48], see Fig. 2g. Both studies had some concerns in overall risk of bias. The random-effects model showed a significant mean difference in dual-task cost on gait speed of − 8.75% (95% CI-14.57, − 2.92) in favor of the motor–cognitive training in contrast to active control groups.

#### Dual-task reaction time

Two studies were included in the meta-analysis for the outcome reaction times of the cognitive task during dual-tasking [40, 53], see Fig. 2h. The random-effects model was not significant (*p* = 0.47) regarding any mean difference in reaction time between motor–cognitive training and control.

#### Timed Up and Go cognitive

Three studies were included in the meta-analysis for the outcome TUG cog [38, 47, 49], see Fig. 2i. Regarding overall risk of bias, two of the studies were assessed as having high risk of bias, and one study as some concerns. The random-effects model was not significant (*p* = 0.17) regarding any mean difference in performance between motor–cognitive training and control.

### Sensitivity analysis

The results of the sensitivity analyses showed a significant training effect on TUG cog using a fixed-effects model, but not using a random-effects model (the fixed-effects model showed a significant mean difference in TUG cog of − 2.16 s (95% CI − 4.05, − 0.27) in favor of the motor–cognitive training). Regarding time spent in double support (%), the fixed-effects model showed significant mean difference (− 1.19% (95% CI − 2.44, 0.05)), but the random-effects model did not. The sensitivity analyses further showed a significant mean difference in dual-task cadence with passive controls included [38], but not significant without (*p* = 0.07). No other differences between random and fixed-effects models, or with/without passive control groups were found. See Online Resource 4, Tables 2 and 3, for a detailed outline of the sensitivity analyses.

### Risk of bias

**Fig. 3:**
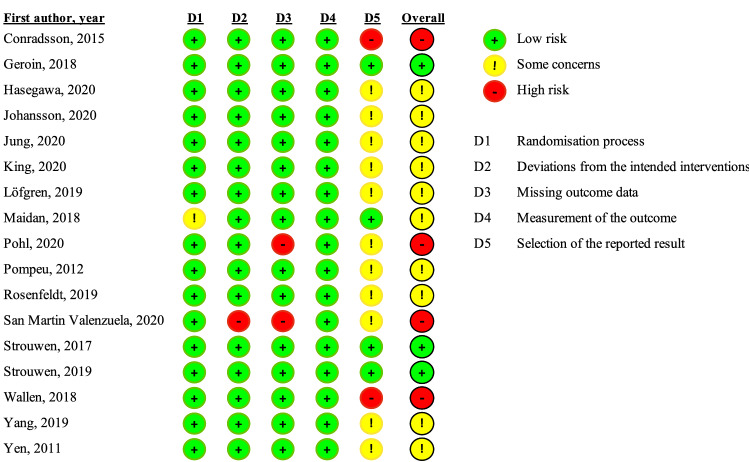
Risk of bias of included studies

We used the RoB2 tool to assess risk of bias for each of the included reports. A summary of the assessments is provided in Fig. 3. A majority of the reports (10/17) were considered to have some concerns in terms of overall risk of bias, and four reports were assessed as having a high overall risk of bias. In all but three of the reports assessed as either high risk of bias or some concerns, the overall risk of bias was driven by the domain pertaining to selection of the reported results. A lack of preregistered or published analysis plan was the primary reason for raised concern regarding selection of the reported results.

### Certainty of evidence

Compared with no training or training without elements of dual-tasking, high certainty evidence suggests that motor–cognitive training increases dual-task gait speed, dual-task cadence, and dual-task step length, and decreases dual-task cost on gait speed (Table 3).

**Table 3:** GRADE evidence profile

Certainty assessment							No. of patients		Effect	Certainty
No. of studies	Study design	Risk of bias	Inconsistency	Indirectness	Imprecision	Other considerations	Motor–cognitive	Control	Absolute (95% CI)	
*Dual-task gait speed (m/s)*										
8	Randomised trials	Not serious	Not serious	Not serious	Not serious	None	223	223	MD 0.12 higher (0.08 higher to 0.17 higher)	⨁⨁⨁⨁ High
*Dual-task cadence (steps/min)*										
5	Randomised trials	Not serious	Not serious	Not serious	Not serious	None	142	142	MD 2.91 higher (0.08 higher to 5.73 higher)	⨁⨁⨁⨁ High
*Dual-task stride length (cm)*										
6	Randomised trials	Not serious	Not serious	Not serious	Not serious	None	206	208	MD 10.12 higher (4.86 higher to 15.38 higher)	⨁⨁⨁⨁ High
*Dual-task stride length SD*										
2	Randomised trials	Not serious	Not serious	Not serious	Not serious	None	103	109	MD 0.38 lower (1.29 lower to 0.53 higher)	⨁⨁⨁⨁ High
*Dual-task stride time SD*										
2	Randomised trials	Not serious	Not serious	Not serious	Not serious	None	103	109	MD 0.01 lower (0.02 lower to 0.01 higher)	⨁⨁⨁⨁ High
*Dual-task double support (%)*										
3	Randomised trials	Not serious	Serious^a^	Not serious	Not serious	None	107	113	MD 1.63 lower (3.76 lower to 0.49 higher)	⨁⨁⨁◯ Moderate
*Dual-task cost on gait speed (%)*										
2	Randomised trials	Not serious	Not serious	Not serious	Not serious	None	50	48	MD 8.75 lower (14.57 lower to 2.92 lower)	⨁⨁⨁⨁ High
*Dual-task reaction time (ms)*										
2	Randomised trials	Not serious	Serious^b^	Not serious	Serious^c^	None	69	78	MD 24.91 higher (43.02 lower to 92.84 higher)	⨁⨁◯◯ Low
*Timed Up and Go Cognitive (sec)*										
3	Randomised trials	Serious^d^	Serious^b^	Not serious	Not serious	None	77	64	MD 1.81 lower (4.38 lower to 0.75 higher)	⨁⨁◯◯ Low

*CI* confidence interval, *MD* mean difference

Explanations

^a^Moderate heterogeneity (48%). Wide variance of point estimates

^b^Wide variance of point estimates across studies

^c^Wide confidence interval around the estimate of the effect

^d^Two of three studies had high overall risk of bias

## Discussion

The aim of this systematic review was to establish the current evidence on the effects of motor–cognitive training on dual-task performance in people with PD. To the best of our knowledge, this is the first systematic review and meta-analysis providing evidence that people with PD can improve their dual-task abilities through motor–cognitive training. The results show that the spatiotemporal gait parameters speed, cadence and stride length increased, while dual-task cost on gait speed decreased, in comparison to passive and/or active controls. No effects were shown on measures of gait variability or percentage of time spent in double support. Results regarding TUG cog were conflicting, with the fixed-effects model significant and the random-effects model not.

Gait speed is an important biomarker of mobility and is widely accepted as the sixth vital sign [55]. Encouragingly, but in contrast to the one previous meta-analysis investigating the effect of motor–cognitive training on dual-task gait speed [22], we did find an effect compared to controls. Although the analyses do not disclose the mean increase in gait speed or contrast the results to single-task gait speed, the results are the first to show that motor–cognitive training can impact bradykinetic dual-task gait. Speed, step length and cadence are highly interrelated and so it is unsurprising that improvements in respective gait parameter follow a similar pattern. The clinically meaningful difference in single-task gait speed in PD ranges from 0.06 m/s (small effect) to 0.22 m/s (large effect) [55], but no such cut-offs are currently available for dual-task gait speed. To what extent the cut-offs can help the interpretation of differences in dual-task gait speed is unclear. Previous research have shown that although single and dual-task gait speeds are highly related [57], the latter is also associated with, for example, executive function [57] and functional balance [58]. The analyses did not indicate any post-intervention, across group differences in gait variability. Although these two meta-analyses are based on two studies only, the results are disconcerting as gait variability is associated with both gait automaticity [59] and fall risk [60].

According to a survey of physiotherapists working with PD patients in Sweden, the most commonly used standardized measurement tool was the TUG test (used by ≥ 97%) [61]. Both the TUG [62] and the TUG with an added cognitive task (TUG cog) [63] can also help identify individuals with PD with a high or low risk of falls. The result of this study showed that the group participating in a motor–cognitive intervention took in mean 2.6 fewer seconds to complete the TUG cog compared to controls (using a fixed-effects model). This may be of clinical importance as deterioration of gait under dual-task straight walking have not shown an association to prospective falls [62]. The TUG test presumably mirrors everyday indoor movements to a larger extent than straight walking tests. Evaluating dual-task performance after motor–cognitive interventions is important, but perhaps a test of straight walking is insufficient if we also want to predict whether the training can affect fall risk in a PD population.

Participants in the studies included in this review typically had mild to moderate disease severity (mean H&Y 2.0). Although this is reflective of PD exercise trials in general, this mild-moderate sample diminishes the ability to understand how motor–cognitive training affects de novo PD or people with severe disease severity. Interestingly, responder analyses from two of the included studies indicate that those benefiting most from motor–cognitive training were people who at baseline had higher global cognition [46], lower dual-task gait speed [46, 50], and took longer time to complete the TUG test [50]. In PD, higher levels of cognitive function, and especially episodic memory and attention, is associated with a faster motor learning acquisition rate [64]. This reliance on episodic memory for motor learning in PD has been suggested as a cognitive compensatory strategy [64], and could partly explain why preserved cognition could be critical for better outcomes after motor–cognitive training. The combined interpretation of findings from these studies suggests that people with impaired motor performance but who have preserved cognition, may be the most suitable target group for motor–cognitive training.

Exploring which type and dose of motor–cognitive training that has the best effect on dual-task performance is beyond the scope of this systematic review. As the field progresses and more high-quality trials are published, it will, however, be of great importance to update and perform such analyses. The interventions described in this review varied in nature, dose, and setting which may have contributed to a clinical heterogeneity. As most included studies had not reported on the amount of time per session spent dual-tasking it is also impossible to infer what proportion of dual-tasking is sufficient. For future studies, we recommend that information regarding the mean individual training dose is included. Data from future studies should provide sufficient power to perform meta-analyses in which subgroup analyses regarding different dual-task training paradigms and intervention doses can be calculated.

There was an overall lack of reporting of performance on the cognitive task in the studies. Such information is crucial for several reasons and should always be considered when designing and/or deciding on a suitable assessment method of dual-tasking. Using standardized and reliability tested cognitive tasks such as the digit span or auditory Stroop [65] allows researchers to evaluate performance on both the motor task and the cognitive task. By doing so, the interpretation of a dual-task gait analyses can be better nuanced and reveal patterns of postural strategies and prioritization.

In the included studies, there was an overall lack of a preregistered or published analysis plan causing concerns during risk of bias assessment in the domain pertaining to selection of the reported results. With the rapid acceleration of open science over the last decade the practice of preregistration has undoubtedly increased. However, several studies in this review were published before such approaches were custom or advocated.

Whereas previous reviews on motor–cognitive training in PD have investigated the general effects on e.g., gait and balance, this is the first study focusing on actual dual-task performance. Although potential transfer effects are interesting, we believe that our novel findings of task-specific effects on dual-task performance after motor–cognitive training in PD is of even higher clinical relevance. Collating all available evidence on a certain topic does however not come without challenges. The search strategy, although striving to be broad in scope, may have failed to identify suitable motor–cognitive interventions if the authors had not described them as such, or as addressing dual-task components. We did however also perform manual searches of the reference lists as well as checking registers for ongoing trials. A total of only 11 studies were included which ultimately limited the ability to perform subgroup analyses. Our results can, therefore, not reveal for example whether people in early versus advanced disease stages, or individuals with or without freezing of gait, benefit differently from motor–cognitive training. A further limitation to this review is that the impact of cognitive state cannot be defined, due to the fact that the cognitive profile of patients is poorly or not described in most studies. Future studies should, therefore, report information on whether study participants suffered from cognitive dysfunction (i.e., subjective cognitive decline, mild cognitive impairment, or dementia) and analyze in which way cognitive state is related to motor-cognitive training response. Finally, the impact of sociodemographic factors including age, education, and sex should be considered in future research. With the rapid advancement in the field and several ongoing trials focusing on motor–cognitive interventions, future systematic reviews will have the opportunity to explore these issues.

This is the first systematic review to show that motor–cognitive interventions as compared with no training or training without elements of dual-tasking have the ability to improve various spatiotemporal aspects of dual-task gait in people with PD. As more studies become available for meta-analysis, future research should focus on discerning who benefits most from this type of intervention, as well as exploring knowledge gaps concrening optimal dose and approach.

## Supplementary Information

Below is the link to the electronic supplementary material.

Supplementary file1 (DOCX 26 KB)

Supplementary file2 (DOCX 359 KB)

Supplementary file3 (DOCX 25 KB)

Supplementary file4 (DOCX 18 KB)

## Data Availability

Data can be made available upon reasonable request.

## References

[1] McIsaac TL, Lamberg EM, Muratori LM (2015). Building a framework for a dual task taxonomy. Biomed Res Int.

[2] Amboni M, Barone P, Hausdorff JM (2013). Cognitive contributions to gait and falls: evidence and implications. Mov Disord.

[3] Al-Yahya E, Dawes H, Smith L, Dennis A, Howells K, Cockburn J (2011). Cognitive motor interference while walking: a systematic review and meta-analysis. Neurosci Biobehav Rev.

[4] Plotnik M, Giladi N, Hausdorff JM (2009). Bilateral coordination of gait and Parkinson's disease: the effects of dual tasking. J Neurol Neurosurg Psychiatry.

[5] Plotnik M, Dagan Y, Gurevich T, Giladi N, Hausdorff JM (2011). Effects of cognitive function on gait and dual tasking abilities in patients with Parkinson's disease suffering from motor response fluctuations. Exp Brain Res.

[6] Lord S, Rochester L, Hetherington V, Allcock LM, Burn D (2010). Executive dysfunction and attention contribute to gait interference in 'off' state Parkinson's disease. Gait Posture.

[7] Yogev-Seligmann G, Giladi N, Gruendlinger L, Hausdorff JM (2013). The contribution of postural control and bilateral coordination to the impact of dual tasking on gait. Exp Brain Res.

[8] Rochester L, Galna B, Lord S, Burn D (2014). The nature of dual-task interference during gait in incident Parkinson's disease. Neuroscience.

[9] Stegemoller EL, Wilson JP, Hazamy A, Shelley MC, Okun MS, Altmann LJ, Hass CJ (2014). Associations between cognitive and gait performance during single- and dual-task walking in people with Parkinson disease. Phys Ther.

[10] Penko AL, Streicher MC, Koop MM, Dey T, Rosenfeldt AB, Bazyk AS, Alberts JL (2018). Dual-task interference disrupts Parkinson's gait across multiple cognitive domains. Neuroscience.

[11] Wild LB, de Lima DB, Balardin JB, Rizzi L, Giacobbo BL, Oliveira HB, Argimon IID, Peyre-Tartaruga LA, Rieder CRM, Bromberg E (2013). Characterization of cognitive and motor performance during dual-tasking in healthy older adults and patients with Parkinson's disease. J Neurol.

[12] Alcock L, Galna B, Lord S, Rochester L (2016). Characterisation of foot clearance during gait in people with early Parkinson's disease: deficits associated with a dual task. J Biomech.

[13] Gassner H, Marxreiter F, Steib S, Kohl Z, Schlachetzki JCM, Adler W, Eskofier BM, Pfeifer K, Winkler J, Klucken J (2017). Gait and cognition in Parkinson's disease: cognitive impairment is inadequately reflected by gait performance during dual task. Front Neurol.

[14] Johansson H, Ekman U, Rennie L, Peterson DS, Leavy B, Franzen E (2021). Dual-task effects during a motor-cognitive task in Parkinson's disease: patterns of prioritization and the influence of cognitive status. Neurorehabil Neural Repair.

[15] Lord SR, Bindels H, Ketheeswaran M, Brodie MA, Lawrence AD, Close JCT, Whone AL, Ben-Shlomo Y, Henderson EJ (2020). Freezing of gait in people with parkinson's disease: nature, occurrence, and risk factors. J Parkinsons Dis.

[16] Jones D, Rochester L, Birleson A, Hetherington V, Nieuwboer A, Willems AM, Van Wegen E, Kwakkel G (2008). Everyday walking with Parkinson's disease: understanding personal challenges and strategies. Disabil Rehabil.

[17] Johansson H, Franzen E, Skavberg Roaldsen K, Hagstromer M, Leavy B (2019). Controlling the uncontrollable: perceptions of balance in people with Parkinson disease. Phys Ther.

[18] Wu T, Hallett M, Chan P (2015). Motor automaticity in Parkinson's disease. Neurobiol Dis.

[19] Fitts PM, Posner MI (1967). Human performance.

[20] Wu T, Chan P, Hallett M (2010). Effective connectivity of neural networks in automatic movements in Parkinson's disease. Neuroimage.

[21] Wajda DA, Mirelman A, Hausdorff JM, Sosnoff JJ (2017). Intervention modalities for targeting cognitive-motor interference in individuals with neurodegenerative disease: a systematic review. Expert Rev Neurother.

[22] Li Z, Wang T, Liu H, Jiang Y, Wang Z, Zhuang J (2020). Dual-task training on gait, motor symptoms, and balance in patients with Parkinson's disease: a systematic review and meta-analysis. Clin Rehabil.

[23] Radder DLM, Silva L, de Lima A, Domingos J, Keus SHJ, van Nimwegen M, Bloem BR, de Vries NM (2020). Physiotherapy in Parkinson's disease: a meta-analysis of present treatment modalities. Neurorehabil Neural Repair.

[24] Strouwen C, Molenaar E, Munks L, Keus SHJ, Bloem BR, Rochester L, Nieuwboer A (2015). Dual tasking in Parkinson's disease: should we train hazardous behavior?. Expert Rev Neurother.

[25] Domellöf ME, Ekman U, Forsgren L, Elgh E (2015). Cognitive function in the early phase of Parkinson's disease, a five-year follow-up. Acta Neurol Scand.

[26] Zhang WS, Gao C, Tan YY, Chen SD (2021). Prevalence of freezing of gait in Parkinson's disease: a systematic review and meta-analysis. J Neurol.

[27] Cosgrove J, Alty JE, Jamieson S (2015). Cognitive impairment in Parkinson's disease. Postgrad Med J.

[28] Ali N, Tian H, Thabane L, Ma J, Wu H, Zhong Q, Gao Y, Sun C, Zhu Y, Wang T (2022). The effects of dual-task training on cognitive and physical functions in older adults with cognitive impairment; a systematic review and meta-analysis. J Prev Alzheimers Dis.

[29] Li Q, Gong B, Zhao Y, Wu C (2022). Effect of exercise cognitive combined training on physical function in cognitively healthy older adults: a systematic review and meta-analysis. J Aging Phys Act.

[30] Rieker JA, Reales JM, Muiños M, Ballesteros S (2022). The effects of combined cognitive-physical interventions on cognitive functioning in healthy older adults: a systematic review and multilevel meta-analysis. Front Hum Neurosci.

[31] Ouzzani M, Hammady H, Fedorowicz Z, Elmagarmid A (2016). Rayyan-a web and mobile app for systematic reviews. Syst Rev.

[32] Ouzzani M, Hammady H, Fedorowicz Z, Elmagarmid A (2016). Rayyan-a web and mobile app for systematic reviews. Syst Rev.

[33] Maidan I, Nieuwhof F, Bernad-Elazari H, Bloem BR, Giladi N, Hausdorff JM, Claassen J, Mirelman A (2018). Evidence for differential effects of 2 forms of exercise on prefrontal plasticity during walking in Parkinson's disease. Neurorehabil Neural Repair.

[34] Pompeu JE, Mendes FA, Silva KG, Lobo AM, Oliveira Tde P, Zomignani AP, Piemonte ME (2012). Effect of Nintendo Wii™-based motor and cognitive training on activities of daily living in patients with Parkinson's disease: a randomised clinical trial. Physiotherapy.

[35] King LA, Mancini M, Smulders K, Harker G, Lapidus JA, Ramsey K, Carlson-Kuhta P, Fling BW, Nutt JG, Peterson DS, Horak FB (2020). Cognitively challenging agility boot camp program for freezing of gait in Parkinson disease. Neurorehabil Neural Repair.

[36] Jung SH, Hasegawa N, Mancini M, King LA, Carlson-Kuhta P, Smulders K, Peterson DS, Barlow N, Harker G, Morris R, Lapidus J, Nutt JG, Horak FB (2020). Effects of the agility boot camp with cognitive challenge (ABC-C) exercise program for Parkinson's disease. npj Parkinsons Dis.

[37] Hasegawa N, Shah VV, Harker G, Carlson-Kuhta P, Nutt JG, Lapidus JA, Jung SH, Barlow N, King LA, Horak FB, Mancini M (2020). Responsiveness of objective vs. clinical balance domain outcomes for exercise intervention in Parkinson's disease. Front Neurol.

[38] Conradsson D, Lofgren N, Nero H, Hagstromer M, Stahle A, Lokk J, Franzen E (2015). The effects of highly challenging balance training in elderly with Parkinson's disease: a randomized controlled trial. Neurorehabil Neural Repair.

[39] Wallen MB, Hagstromer M, Conradsson D, Sorjonen K, Franzen E (2018). Long-term effects of highly challenging balance training in Parkinson's disease-a randomized controlled trial. Clin Rehabil.

[40] Strouwen C, Molenaar E, Munks L, Keus SHJ, Zijlmans JCM, Vandenberghe W, Bloem BR, Nieuwboer A (2017). Training dual tasks together or apart in Parkinson's disease: results from the DUALITY trial. Mov Disord.

[41] Review Manager (RevMan) (2020) In: The Cochrane Collaboration

[42] Higgins JPT, Thomas J, Chandler J, Cumpston M, Li T, Page MJ, Welch VA (eds) (2022) Cochrane handbook for systematic reviews of interventions version 6.3 (updated February 2022). Cochrane. Available from www.training.cochrane.org/handbook 10.1002/14651858.ED000142 PMC10284251 31643080

[43] GRADEpro GDT: GRADEpro Guideline Development Tool [Software] (2022) In: McMaster University and Evidence Prime

[44] Page MJ, McKenzie JE, Bossuyt PM, Boutron I, Hoffmann TC, Mulrow CD, Shamseer L, Tetzlaff JM, Akl EA, Brennan SE, Chou R, Glanville J, Grimshaw JM, Hróbjartsson A, Lalu MM, Li T, Loder EW, Mayo-Wilson E, McDonald S, McGuinness LA, Stewart LA, Thomas J, Tricco AC, Welch VA, Whiting P, Moher D (2021). The PRISMA 2020 statement: an updated guideline for reporting systematic reviews. BMJ (Online).

[45] Geroin C, Nonnekes J, de Vries NM, Strouwen C, Smania N, Tinazzi M, Nieuwboer A, Bloem BR (2018). Does dual-task training improve spatiotemporal gait parameters in Parkinson's disease?. Parkinsonism Relat Disord.

[46] Strouwen C, Molenaar E, Munks L, Broeder S, Ginis P, Bloem BR, Nieuwboer A, Heremans E (2019). Determinants of dual-task training effect size in Parkinson disease: who will benefit most?. J Neurol Phys Ther.

[47] Pohl P, Wressle E, Lundin F, Enthoven P, Dizdar N (2020). Group-based music intervention in Parkinson's disease—findings from a mixed-methods study. Clin Rehabil.

[48] Yang YR, Cheng SJ, Lee YJ, Liu YC, Wang RY (2019). Cognitive and motor dual task gait training exerted specific training effects on dual task gait performance in individuals with Parkinson's disease: a randomized controlled pilot study. PLoS ONE.

[49] Johansson H, Freidle M, Ekman U, Schalling E, Leavy B, Svenningsson P, Hagstromer M, Franzen E (2020). Feasibility aspects of exploring exercise-induced neuroplasticity in Parkinson's disease: a pilot randomized controlled trial. Parkinsons Dis.

[50] Lofgren N, Conradsson D, Rennie L, Moe-Nilssen R, Franzen E (2019). The Effects of integrated single- and dual-task training on automaticity and attention allocation in parkinson's disease: a secondary analysis from a randomized trial. Neuropsychology.

[51] Rosenfeldt AB, Penko AL, Streicher MC, Zimmerman NM, Koop MM, Alberts JL (2019). Improvements in temporal and postural aspects of gait vary following single- and multi-modal training in individuals with Parkinson's disease. Parkinsonism Relat Disord.

[52] San Martín Valenzuela C, Moscardó LD, López-Pascual J, Serra-Añó P, Tomás JM (2020). Effects of dual-task group training on gait, cognitive executive function, and quality of life in people with parkinson disease: results of randomized controlled DUALGAIT trial. Arch Phys Med Rehabil.

[53] Yen CY, Lin KH, Hu MH, Wu RM, Lu TW, Lin CH (2011). Effects of virtual reality-augmented balance training on sensory organization and attentional demand for postural control in people with Parkinson disease: a randomized controlled trial. Phys Ther.

[54] Fritz S, Lusardi M (2009). White paper: "walking speed: the sixth vital sign". J Geriatr Phys Ther.

[55] Hass CJ, Bishop M, Moscovich M, Stegemoller EL, Skinner J, Malaty IA, Wagle Shukla A, McFarland N, Okun MS (2014). Defining the clinically meaningful difference in gait speed in persons with Parkinson disease. J Neurol Phys Ther.

[56] Strouwen C, Molenaar E, Keus SHJ, Munks L, Heremans E, Vandenberghe W, Bloem BR, Nieuwboer A (2016). Are factors related to dual-task performance in people with Parkinson's disease dependent on the type of dual task?. Parkinsonism Relat Disord.

[57] Christofoletti G, McNeely ME, Campbell MC, Duncan RP, Earhart GM (2016). Investigation of factors impacting mobility and gait in Parkinson disease. Hum Mov Sci.

[58] Gilat M, Bell PT, Martens KAE, Georgiades MJ, Hall JM, Walton CC, Lewis SJG, Shine JM (2017). Dopamine depletion impairs gait automaticity by altering cortico-striatal and cerebellar processing in Parkinson's disease. Neuroimage.

[59] Henderson EJ, Lord SR, Brodie MA, Gaunt DM, Lawrence AD, Close JC, Whone AL, Ben-Shlomo Y (2016). Rivastigmine for gait stability in patients with Parkinson's disease (ReSPonD): a randomised, double-blind, placebo-controlled, phase 2 trial. Lancet Neurol.

[60] Conradsson D, Leavy B, Hagstromer M, Nilsson MH, Franzen E (2017). Physiotherapy for Parkinson's disease in Sweden: provision, expertise, and multi-professional collaborations. Mov Disorders Clin Pract.

[61] Smulders K, Esselink RA, Weiss A, Kessels RP, Geurts AC, Bloem BR (2012). Assessment of dual tasking has no clinical value for fall prediction in Parkinson's disease. J Neurol.

[62] Vance RC, Healy DG, Galvin R, French HP (2015). Dual tasking with the timed "up & go" test improves detection of risk of falls in people with Parkinson disease. Phys Ther.

[63] Lofgren N, Conradsson D, Joseph C, Leavy B, Hagstromer M, Franzen E (2019). Factors associated with responsiveness to gait and balance training in people with Parkinson disease. J Neurol Phys Ther.

[64] Chung YC, Fisher BE, Finley JM, Kim A, Petkus AJ, Schiehser DM, Jakowec MW, Petzinger GM (2021). Cognition and motor learning in a Parkinson's disease cohort: importance of recall in episodic memory. NeuroReport.

[65] Strouwen C, Molenaar E, Keus SHJ, Munks L, Bloem BR, Nieuwboer A (2016). Test-retest reliability of dual-task outcome measures in people with Parkinson disease. Phys Ther.

