# Supplementary material for: Effects of motor–cognitive training on dual-task performance in people with Parkinson’s disease: a systematic review and meta-analysis
Source: J Neurol. 2023 Feb 23;270(6):2890–907. doi: 10.1007/s00415-023-11610-8 (PMC10188503; doi:10.1007/s00415-023-11610-8)
Supplement: Supplementary file 1 — Supplementary file1 (DOCX 26 KB) [file 415_2023_11610_MOESM1_ESM.docx]

## Online Resource 1. Systematic search strategies

Medline

| Interface: Ovid MEDLINE(R) and Epub Ahead of Print, In-Process & Other Non-Indexed Citations and Daily  Date of Search: 28 September 2021  Number of hits: 1,039  Comment: In Ovid, two or more words are automatically searched as phrases; i.e. no quotation marks are needed | Field labels   - exp/ = exploded MeSH term - / = non exploded MeSH term - .ti,ab,kf. = title, abstract and author keywords - adjx = within x words, regardless of order - * = truncation of word for alternate endings |
| --- | --- |
| Database(s): **Ovid MEDLINE(R) and Epub Ahead of Print, In-Process, In-Data-Review & Other Non-Indexed Citations and Daily**1946 to September 27, 2021 Search Strategy:   \| **#** \| **Searches** \| **Results** \| \| --- \| --- \| --- \| \| 1 \| Parkinson Disease/ \| 72309 \| \| 2 \| parkinson*.ti,ab,kf. \| 129218 \| \| 3 \| 1 or 2 \| 136853 \| \| 4 \| Virtual Reality/ \| 3353 \| \| 5 \| (cognit* task* or divided attention or duality trail or multitask* or multi* task*).ti,ab,kf. \| 16150 \| \| 6 \| ((multi-component or multi-dimension* or multi-modal) adj3 training).ti,ab,kf. \| 111 \| \| 7 \| (cognit* adj3 motor adj3 (interfere* or task* or training)).ti,ab,kf. \| 1143 \| \| 8 \| ((cognit* or motor) adj2 task* adj2 interfere*).ti,ab,kf. \| 157 \| \| 9 \| ((attention-demanding or concurrent or dual or second or secondary or simultaneous*) adj3 task*).ti,ab,kf. \| 11638 \| \| 10 \| ((augmented or virtual) adj2 (environment* or realit*)).ti,ab,kf. \| 17917 \| \| 11 \| or/4-10 \| 44477 \| \| 12 \| 3 and 11 \| 1057 \| \| 13 \| limit 12 to (english or german or swedish) \| 1039 \| | |

Cochrane Central Register of Controlled Trials

| Interface: Wiley  Date of Search: 27 September 2021  Number of hits: 485 | Field labels   - ti,ab,kw = title, abstract and author keywords - NEAR/x = within x words, regardless of order - * = truncation of word for alternate endings |
| --- | --- |
| \| ID \| Search \| Hits \| \| --- \| --- \| --- \| \| #1 \| MeSH descriptor: [Parkinson Disease] this term only \| 4444 \| \| #2 \| parkinson*:ti,ab,kw \| 11449 \| \| #3 \| #1 or #2 \| 11449 \| \| #4 \| MeSH descriptor: [Virtual Reality] this term only \| 328 \| \| #5 \| ((cognit* NEXT task*) OR "divided attention" OR "duality trail" OR multitask* OR (multi* NEXT task*)):ti,ab,kw \| 2502 \| \| #6 \| (("multi-component" OR ("multi" NEXT dimension*) OR "multi-modal") NEAR/3 "training"):ti,ab,kw \| 1858 \| \| #7 \| (cognit* NEAR/3 "motor" NEAR/3 (interfere* OR task* OR "training")):ti,ab,kw \| 363 \| \| #8 \| ((cognit* OR "motor") NEAR/2 task* NEAR/2 interfere*):ti,ab,kw \| 22 \| \| #9 \| (("attention-demanding" OR "concurrent" OR "dual" OR "second" OR "secondary" OR simultaneous*) NEAR/3 task*):ti,ab,kw \| 2151 \| \| #10 \| (("augmented" OR "virtual") NEAR/2 (environment* OR realit*)):ti,ab,kw \| 4460 \| \| #11 \| #4 or #5 or #6 or #7 or #8 or #9 or #10 \| 10489 \| \| #12 \| #3 and #11 \| 487 (485 in Trials) \| | |

Web of Science Core Collection

| Interface: Clarivate Analytics  Date of Search: 28 September 2021  Number of hits: 1,787 | Field labels   - TS/Topic = title, abstract, author keywords and Keywords Plus - NEAR/x = within x words, regardless of order - * = truncation of word for alternate endings   Note: sometimes “quotation marks” are needed for single search terms to avoid automatic term mapping (lemmatization). |
| --- | --- |
| \| **Set** \| \| **Results** \|  \| \| --- \| --- \| --- \| --- \| \| # 10 \| [**1,787**](http://apps.webofknowledge.com/summary.do;jsessionid=35FC5061D12A358C9242CEAF405FE8DB?product=WOS&doc=1&qid=10&SID=E2DEuwubljsb3hUJBZZ&search_mode=CombineSearches&update_back2search_link_param=yes) \| \| #8 AND #1  **Refined by:** **LANGUAGES:** ( ENGLISH OR GERMAN )  *Indexes=SCI-EXPANDED, SSCI, A&HCI, ESCI Timespan=All years* \| \| # 9 \| [**1,813**](http://apps.webofknowledge.com/summary.do;jsessionid=35FC5061D12A358C9242CEAF405FE8DB?product=WOS&doc=1&qid=9&SID=E2DEuwubljsb3hUJBZZ&search_mode=CombineSearches&update_back2search_link_param=yes) \| \| #8 AND #1  *Indexes=SCI-EXPANDED, SSCI, A&HCI, ESCI Timespan=All years* \| \| # 8 \| [**86,342**](http://apps.webofknowledge.com/summary.do;jsessionid=35FC5061D12A358C9242CEAF405FE8DB?product=WOS&doc=1&qid=8&SID=E2DEuwubljsb3hUJBZZ&search_mode=CombineSearches&update_back2search_link_param=yes) \| \| #7 OR #6 OR #5 OR #4 OR #3 OR #2  *Indexes=SCI-EXPANDED, SSCI, A&HCI, ESCI Timespan=All years* \| \| # 7 \| [**46,086**](http://apps.webofknowledge.com/summary.do;jsessionid=35FC5061D12A358C9242CEAF405FE8DB?product=WOS&doc=1&qid=7&SID=E2DEuwubljsb3hUJBZZ&search_mode=GeneralSearch&update_back2search_link_param=yes) \| \| **TOPIC:** ((("augmented" OR "virtual") NEAR/1 (environment* OR realit*) ))  *Indexes=SCI-EXPANDED, SSCI, A&HCI, ESCI Timespan=All years* \| \| # 6 \| [**16,909**](http://apps.webofknowledge.com/summary.do;jsessionid=35FC5061D12A358C9242CEAF405FE8DB?product=WOS&doc=1&qid=6&SID=E2DEuwubljsb3hUJBZZ&search_mode=GeneralSearch&update_back2search_link_param=yes) \| \| **TOPIC:** ((("attention-demanding" OR "concurrent" OR "dual" OR "second" OR "secondary" OR simultaneous*) NEAR/2 task*))  *Indexes=SCI-EXPANDED, SSCI, A&HCI, ESCI Timespan=All years* \| \| # 5 \| [**166**](http://apps.webofknowledge.com/summary.do;jsessionid=35FC5061D12A358C9242CEAF405FE8DB?product=WOS&doc=1&qid=5&SID=E2DEuwubljsb3hUJBZZ&search_mode=GeneralSearch&update_back2search_link_param=yes) \| \| **TOPIC:** (((cognit* OR "motor") NEAR/1 task* NEAR/1 interfere*))  *Indexes=SCI-EXPANDED, SSCI, A&HCI, ESCI Timespan=All years* \| \| # 4 \| [**1,262**](http://apps.webofknowledge.com/summary.do;jsessionid=35FC5061D12A358C9242CEAF405FE8DB?product=WOS&doc=1&qid=4&SID=E2DEuwubljsb3hUJBZZ&search_mode=GeneralSearch&update_back2search_link_param=yes) \| \| **TOPIC:** ((cognit* NEAR/2 "motor" NEAR/2 (interfere* OR task* OR "training") ))  *Indexes=SCI-EXPANDED, SSCI, A&HCI, ESCI Timespan=All years* \| \| # 3 \| [**171**](http://apps.webofknowledge.com/summary.do;jsessionid=35FC5061D12A358C9242CEAF405FE8DB?product=WOS&doc=1&qid=3&SID=E2DEuwubljsb3hUJBZZ&search_mode=GeneralSearch&update_back2search_link_param=yes) \| \| **TOPIC:** ((("multi-component" OR "multi-dimension*" OR "multi-modal") NEAR/2 "training"))  *Indexes=SCI-EXPANDED, SSCI, A&HCI, ESCI Timespan=All years* \| \| # 2 \| [**26,222**](http://apps.webofknowledge.com/summary.do;jsessionid=35FC5061D12A358C9242CEAF405FE8DB?product=WOS&doc=1&qid=2&SID=E2DEuwubljsb3hUJBZZ&search_mode=GeneralSearch&update_back2search_link_param=yes) \| \| **TOPIC:** (("cognit* task*" OR "divided attention" OR "duality trail" OR multitask* OR "multi* task*") )  *Indexes=SCI-EXPANDED, SSCI, A&HCI, ESCI Timespan=All years* \| \| # 1 \| [**188,748**](http://apps.webofknowledge.com/summary.do;jsessionid=35FC5061D12A358C9242CEAF405FE8DB?product=WOS&doc=1&qid=1&SID=E2DEuwubljsb3hUJBZZ&search_mode=GeneralSearch&update_back2search_link_param=yes) \| \| **TOPIC:** (parkinson*)  *Indexes=SCI-EXPANDED, SSCI, A&HCI, ESCI Timespan=All years* \| | |

Cinahl

| Interface: Ebsco  Date of Search: 27 September 2021  Number of hits: 478 | Field labels   - MH+ = exploded Cinahl Heading - MH = non exploded Cinahl Heading - TI = title - AB = abstract - Nx = within x words, regardless of order - * = truncation of word for alternate endings |
| --- | --- |
| \| S12 \| S3 AND S11 \| 478 \| \| --- \| --- \| --- \| \| S11 \| S4 OR S5 OR S6 OR S7 OR S8 OR S9 OR S10 \| 16,527 \|  \| \| S10 \| TI ( (("augmented" OR "virtual") N1 (environment* OR realit*)) ) OR AB ( (("augmented" OR "virtual") N1 (environment* OR realit*)) ) \| 6,540 \|  \| \| S9 \| TI ( (("attention-demanding" OR "concurrent" OR "dual" OR "second" OR "secondary" OR simultaneous*) N2 task*)) ) OR AB ( (("attention-demanding" OR "concurrent" OR "dual" OR "second" OR "secondary" OR simultaneous*) N2 task*)) ) \| 3,369 \| \| S8 \| TI ( ((cognit* OR "motor") N1 task* N1 interfere*)) ) OR AB ( ((cognit* OR "motor") N1 task* N1 interfere*)) ) \| 43 \| \| S7 \| TI ( ((cognit* N2 "motor" N2 (interfere* OR task* OR "training")) ) OR AB ( ((cognit* N2 "motor" N2 (interfere* OR task* OR "training")) ) \| 371 \| \| S6 \| TI ( ((("multi-component" OR "multi-dimension*" OR "multi-modal") N2 "training")) ) OR AB ( ((("multi-component" OR "multi-dimension*" OR "multi-modal") N2 "training") ) \| 64 \| \| S5 \| TI ( (("cognit* task*" OR "divided attention" OR "duality trail" OR multitask* OR "multi* task*") ) ) OR AB ( (("cognit* task*" OR "divided attention" OR "duality trail" OR multitask* OR "multi* task*") ) ) \| 3,933 \| \| S4 \| (MH "Virtual Reality") \| 5,896 \| \| S3 \| S1 OR S2 \| 32,575 \| \| S2 \| TI parkinson* OR AB parkinson* \| 28,899 \| \| S1 \| (MH "Parkinson Disease") \| 23,346 \| | |
|  | |
