# Supplementary material for: Effects of motor–cognitive training on dual-task performance in people with Parkinson’s disease: a systematic review and meta-analysis
Source: J Neurol. 2023 Feb 23;270(6):2890–907. doi: 10.1007/s00415-023-11610-8 (PMC10188503; doi:10.1007/s00415-023-11610-8)
Supplement: Supplementary file 2 — Supplementary file2 (DOCX 359 KB) [file 415_2023_11610_MOESM2_ESM.docx]

## Online Resource 2.

**Fig 1a** Forest plot. Motor-cognitive training vs control. Outcome: dual task cost on stride length

**Fig 1b** Forest plot. Motor-cognitive training vs control. Outcome: dual task accurac

**Fig 1c** Forest plot. Motor-cognitive training vs control. Outcome: dual task cost on accuracy

**Fig 1d** Forest plot. Motor-cognitive training vs control. Outcome: dual task unipedal stance test
