# Supplementary material for: Effects of motor–cognitive training on dual-task performance in people with Parkinson’s disease: a systematic review and meta-analysis
Source: J Neurol. 2023 Feb 23;270(6):2890–907. doi: 10.1007/s00415-023-11610-8 (PMC10188503; doi:10.1007/s00415-023-11610-8)
Supplement: Supplementary file 3 — Supplementary file3 (DOCX 25 KB) [file 415_2023_11610_MOESM3_ESM.docx]

## Online Resource 3.

**Table 1.** Excluded studies with reasons.

| **First author, Year** | **Title** | **Reason for exclusion** |
| --- | --- | --- |
| Albani, 2006 | Motor deficit in virtual environment: A study in Parkinson's disease. | Conference abstract/Poster |
| Alves, 2018 | Nintendo Wii TM Versus Xbox Kinect TM for Assisting People With Parkinson's Disease. | Study design did not meet inclusion criteria |
| Araujo, 2021 | Trunk postural control in seated position on a prototype of unstable chair and the interference of cognitive and motor tasks in individuals with Parkinson's disease. | Conference abstract/Poster |
| Baram, 2001 | Walking on tiles: Virtual reality in closed-loop improves gait in Parkinson's patients. | Study design did not meet inclusion criteria |
| Barboza, 2019 | Physiotherapy Versus Physiotherapy Plus Cognitive Training on Cognition and Quality of Life in Parkinson Disease: Randomized Clinical Trial. | Intervention did not meet inclusion criteria |
| Beck, 2018 | Can Dual Task Walking Improve in Parkinson's Disease After External Focus of Attention Exercise? A Single Blind Randomized Controlled Trial. | Intervention did not meet inclusion criteria |
| Bedeschi Ferrari, 2012 | Gait training associated with executive functions tasks in subjects with Parkinson's disease: improvement of performance and effects in motor learning. | Conference abstract/Poster |
| Bedeschi Ferrari, 2011 | Improvement of gait, functional and cognitive performance in patients with parkinson's disease after gait training associated with executive function tasks. | Conference abstract/Poster |
| Bekkers, 2018 | The effects of treadmill training augmented by virtual reality on postural control and freezing of gait in Parkinson's disease. | Conference abstract/Poster |
| Bekkers, 2020 | Do Patients With Parkinson's Disease With Freezing of Gait Respond Differently Than Those Without to Treadmill Training Augmented by Virtual Reality? | No dual task outcome. |
| Bell, 2017 | Striatal dysfunction during dual-task performance in Parkinson's disease. | Commentary |
| Belton, 2014 | The effect of a balance exercise class on activity limitations in people with parkinson's disease. | Conference abstract/Poster |
| Bueno, 2017 | Comparison of three physical therapy interventions with an emphasis on the gait of individuals with Parkinson's disease | No dual task outcome. |
| Byl, 2009 | Enhancing safe mobility in patients with Parkinson's disease: effect of dual task training during aerobic and moderate exercise. | Conference abstract/Poster |
| Camicioli, 1998 | A dual-task interferes with walking in Parkinson's disease patients with freezing. | Conference abstract/Poster |
| Christofoletti, 2010 | Physical therapy improves the balance of patients with Parkinson's disease: a randomized controlled trial. | Conference abstract/Poster |
| de Melo, 2018 | Effect of virtual reality training on walking distance and physical fitness in individuals with Parkinson's disease. | Intervention did not meet inclusion criteria |
| Evans, 2009 | Walking and talking therapy: Improving cognitive-motor dual-tasking in neurological illness. | Wrong population |
| Feng, 2019 | Virtual Reality Rehabilitation Versus Conventional Physical Therapy for Improving Balance and Gait in Parkinson's Disease Patients: A Randomized Controlled Trial. | No dual-task outcome. Corresponding author contacted without response. |
| Fernandes, 2015 | Effects of dual-task training on balance and executive functions in Parkinson's disease: A pilot study. | No dual-task outcome |
| Ferraz, 2018 | The Effects of Functional Training, Bicycle Exercise, and Exergaming on Walking Capacity of Elderly Patients With Parkinson Disease: A Pilot Randomized Controlled Single-blinded Trial | No dual-task outcome. Corresponding author contacted without response. |
| Franzén, 2014 | Efficacy of a highly challenging and systems-specific balance training program in elderly with Parkinson's disease. | Conference abstract/Poster |
| Gandolfi, 2017 | Virtual Reality Telerehabilitation for Postural Instability in Parkinson's Disease: A Multicenter, Single-Blind, Randomized, Controlled Trial | No dual-task outcome. Corresponding author contacted without response. |
| Germin, 2017 | Are the exergaming effective for improving functional exercise capacity, and quality of life in patients with parkinson's disease? | Conference abstract/Poster |
| Hasmann, 2014 | Single and Dual tasking in High Risk Individuals for Parkinson. | Conference abstract/Poster |
| Hausdorff, 2010 | Added benefits of using virtual reality for improving gait and reducing falls in Parkinson’s disease. | Conference abstract/Poster |
| Hung, 2021 | Dual-task interference on weight-shifting in Parkinson's Disease with freezing of gait and without freezing of gait. | Conference abstract/Poster |
| Isernia, 2020 | Effects of an Innovative Telerehabilitation Intervention for People With Parkinson's Disease on Quality of Life, Motor, and Non-motor Abilities. | Study design did not meet inclusion criteria |
| Ivey*, 2010 | Exercise and Cognitive Training in Parkinson's Disease. | Trial registration |
| Jabre, 2012 | Efficacy of double-task training on gait performance in Parkinson's disease: a randomized, controlled, double-blind study. | Conference abstract/Poster |
| Jenkins, 2007 | Dual task interference: The effects of verbal cognitive tasks on upright postural stability in Parkinson's disease. | Conference abstract/Poster |
| Joseph, 2020 | Predictors of improved balance performance in persons with Parkinson's disease following a training intervention: analysis of data from an effectiveness-implementation trial. | Study design did not meet inclusion criteria |
| Kafle, 2021 | Effect of Wii-Based Motor and Cognitive Training on Activities of Daily Living in Patients with Parkinson's Disease. | Intervention did not meet inclusion criteria |
| King, 2017 | A combined cognitive and motor exercise program for people with Parkinson's disease and Freezing of gait; a pilot study. | Conference abstract/Poster |
| Leavy, 2020 | Outcome Evaluation of Highly Challenging Balance Training for People With Parkinson Disease: a Multicenter Effectiveness-Implementation Study. | Study design did not meet inclusion criteria |
| Lee, 2018 | A virtual reality exercise program improves the balance function and quality of life of patients with Parkinson's disease. | Conference abstract/Poster |
| Lee, 2016 | Virtual-reality balance training with Nintendo-Wii games improves dynamic balance in Parkinson's disease patients. | Conference abstract/Poster |
| Lee, 2019 | Effects of virtual reality exercise program using the sony playstation 2 gaming platform on balance, emotion and quality of life in patients with Parkinson's disease. | Conference abstract/Poster |
| Lee, 2015 | Effect of virtual reality dance exercise on the balance, activities of daily living, and depressive disorder status of Parkinson's disease patients. | Intervention did not meet inclusion criteria |
| Liao, 2015 | Virtual Reality–Based Training to Improve Obstacle-Crossing Performance and Dynamic Balance in Patients With Parkinson’s Disease. | Duplicate |
| Liao, 2015 | Virtual Reality–Based Training to Improve Obstacle-Crossing Performance and Dynamic Balance in Patients With Parkinson’s Disease. | No dual-task outcome. Corresponding author contacted without response. |
| Liao, 2015 | Virtual Reality-Based Wii Fit Training in Improving Muscle Strength, Sensory Integration Ability, and Walking Abilities in Patients with Parkinson's Disease: a Randomized Control Trial. | No dual-task outcome. Corresponding author contacted without response. |
| Lin, 2011 | Effects of virtual reality training on verbal reaction time in subjects with parkinson's disease during functional reach. |  |
| Lopes, 2018 | Is virtual reality really effective in Parkinson's disease? | Commentary |
| Maggio, 2018 | What About the Role of Virtual Reality in Parkinson Disease's Cognitive Rehabilitation? Preliminary Findings From a Randomized Clinical Trial. | Intervention did not meet inclusion criteria |
| Maidan, 2017 | The effects of different modalities of gait training on brain activation in patients with Parkinson's disease: an fMRI study. | Conference abstract/Poster |
| Maidan, 2017 | Disparate effects of training on brain activation in Parkinson disease. | No dual task outcome. |
| Melo, 2018 | Effects of virtual reality training on mobility in individuals with Parkinson's disease. | Duplicate |
| Melo, 2018 | P100 - Effects of virtual reality training on mobility in individuals with Parkinson's disease. | Study design did not meet inclusion criteria |
| Morris, 1999 | Dual task performance in Parkinson's disease. | Conference abstract/Poster |
| Ogundele, 2018 | Comparison of virtual-reality-gaming and activity-based gait and balance training on gait, balance and quality of life in patients with Parkinson's disease. | Conference abstract/Poster |
| Pazzaglia, 2020 | Comparison of virtual reality rehabilitation and conventional rehabilitation in Parkinson's disease: a randomised controlled trial | No dual-task outcome. Corresponding author contacted without response. |
| Pascucci Sande de Souza, 2020 | Effect of motor and cognitive training of the elderly in relation to the ability to perform daily tasks. | Trial registration |
| Penko, 2019 | Multimodal Training Reduces Fall Frequency as Physical Activity Increases in Individuals With Parkinson's Disease. | No dual-task outcome. Corresponding author contacted without response. |
| Perumal, 2016 | Effect of single task training and dual task training on balance in individuals with Idiopathic Parkinson's disease. | Conference abstract/Poster |
| Piementel Piemonte, 2017 | Manual and Verbal guidance during the balance training based in virtual reality is essential for therapeutic results in people with Parkinson's disease: a randomized clinical trial. | Conference abstract/Poster |
| Pimentel Piemonte, 2011 | Improvement of gait, functional and cognitive performance in patients with parkinson's disease after motor and cognitive training. | Conference abstract/Poster |
| PM, 2020 | Virtual Reality für Menschen mit Parkinson. | Commentary |
| Pompeu, 2012 | Functional Improvement in Patients With Parkinson's Disease After Training in Real or Virtual Environment. | Conference abstract/Poster |
| Pompeu, 2011 | Balance improvement in patients with parkinson's disease after motor and cognitive training. |  |
| Pompeu, 2014 | Improvement on gait, functional status and cognition after motor cognitive training in patients with Parkinson’s disease. | Conference abstract/Poster |
| Pompeu, 2012 | Effect of Nintendo WiiBased motor and cognitive training on activities of daily living in patients with Parkinson's disease: a randomised clinical trial. | Duplicate |
| Pompeu, 2012 | Functional improvement in patients with Parkinson's disease after balance and cognitive training in real or virtual environments. | Conference abstract/Poster |
| Pompeu, 2012 | Functional improvement in patients with Parkinson's disease after balance and cognitive training in real or virtual environments. | Trial registration |
| Pompeu, 2012 | Gait improvement in patients with Parkinson's disease after training in real and virtual environments. | Conference abstract/Poster |
| Pompeu, 2016 | Effect of European physiotherapy guideline for Parkinson's disease and Microsoft Kinect® adventures games training on postural control, cognition and quality of life: randomized clinical trial. | Conference abstract/Poster |
| Pompeu, 2014 | Effect of functional training of upper limb in dual task condition in patients with Parkinson’s disease. | Conference abstract/Poster |
| Ponte, 2019 | Changes in gait parameters after a virtual reality protocol (V-Time) in patients with Parkinson's disease. | Withdrawn |
| Pourkhani, 2020 | The Effects of Cognitive and Motor Dual-Task Training on Improvement of Balance, Quality of Life, and Fear of Falls in People with Idiopathic Parkinson's Disease. | No dual-task outcome |
| Rennie, 2020 | Highly challenging balance and gait training for individuals with Parkinson's disease improves pace, rhythm and variability domains of gait - A secondary analysis from a randomized controlled trial. | No dual-task outcome |
| Ribas, 2017 | Effectiveness of exergaming in improving functional balance, fatigue and quality of life in Parkinson's disease: A pilot randomized controlled trial. | No dual-task outcome. Corresponding author contacted without response. |
| Sahu, 2018 | Dual Task Training, Fall, and Functional Independence in Patients with Parkinson's Disease: A Longitudinal Study. | Study design did not meet inclusion criteria |
| Santos, 2019 | Efficacy of the Nintendo Wii combination with Conventional Exercises in the rehabilitation of individuals with Parkinson's disease: A randomized clinical trial. | No dual-task outcome. Corresponding author contacted without response. |
| Sarasso, 2021 | Action Observation and Motor Imagery Improve Dual Task in Parkinson's Disease: a Clinical/fMRI Study. | Intervention did not meet inclusion criteria |
| Sawada, 2020 | Motor and cognitive contributions of dual task walking deficits in people with Parkinson’s disease. | Conference abstract/Poster |
| ShankerTedla, 2017 | Effectiveness of motor task interference during gait in subjects with Parkinson’s disease: a randomized controlled trial. | No dual-task outcome. |
| Shih, 2011 | Effects of virtual reality-augmented sitting balance training on pressure distribution and functional performance in patients with parkinson's disease. | Conference abstract/Poster |
| Shih, 2016 | Effects of a balance-based exergaming intervention using the Kinect sensor on posture stability in individuals with Parkinson's disease: a single-blinded randomized controlled trial. | No dual-task outcome |
| Shook, 2002 | Parkinson's disease patients' performance on motor and cognitive switching tasks. | Conference abstract/Poster |
| Silva, 2019 | Effects of dual-task aquatic exercises on functional mobility, balance and gait of individuals with Parkinson's disease: A randomized clinical trial with a 3-month follow-up. | No dual-task outcome |
| Silva, 2021 | Dual-task intervention based on trail making test: Effects on Parkinson's disease | No dual-task outcome. Corresponding author contacted without response. |
| Smaili, 2019 | Immediate effect of the virtual and augmented reality and neurofunctional physiotherapy on postural control and cognition in patients with Parkinson's disease: a randomized clinical trial. | Conference abstract/Poster |
| Souza, 2013 | The addition of a concurrent task changes postural reactions in individuals with Parkinson's disease. | Conference abstract/Poster |
| Tollár, 2018 | A High-Intensity Multicomponent Agility Intervention Improves Parkinson Patients' Clinical and Motor Symptoms. | Intervention did not meet inclusion criteria |
| Tomo, 2014 | Effects of upper limb functional training in the dual task condition in Parkinson’s disease. | Wrong language |
| Vieira-Yano, 2021 | The Adapted Resistance Training with Instability Randomized Controlled Trial for Gait Automaticity | Intervention did not meet inclusion criteria |
| Weghorst, 1997 | Augmented reality and Parkinson's Disease. | Study design did not meet inclusion criteria |
| Welman, 2018 | Therapist-supervised compared to home-based balance training encourages a 'posture first' strategy during turn-to-sit transitions in individuals with Parkinson's disease. | Conference abstract/Poster |
| Wong-Yu, 2015 | Multi-dimensional balance training programme improves balance and gait performance in people with Parkinson's disease: A pragmatic randomized controlled trial with 12-month follow-up. | Intervention did not meet inclusion criteria |
| Wong-Yu, 2015 | Task- and Context-Specific Balance Training Program Enhances Dynamic Balance and Functional Performance in Parkinsonian Nonfallers: A Randomized Controlled Trial With Six-Month Follow-Up. | Intervention did not meet inclusion criteria |
| Wong-Yu, 2014 | Long-term effects of the hopeful outdoor Parkinson's exercise (HOPE) program on enhancing the dynamic balance and gait performance in people with Parkinson's disease. | Conference abstract/Poster |
| Wong-Yu, 2019 | Multisystem Balance Training Reduces Injurious Fall Risk in Parkinson Disease: A Randomized Trial. | Intervention did not meet inclusion criteria |
| Xu, 2013 | Cognitive tasks influence balance performance in people with Parkinson's disease. | Conference abstract/Poster |
| Yang, 2016 | Home-based virtual reality balance training and conventional balance training in Parkinson's disease: A randomized controlled trial. | No dual-task outcome. Corresponding author contacted without response. |
| Yen, 2011 | Effects of Virtual Reality-Augmented Balance Training on Sensory Organization and Attentional Demand for Postural Control in People With Parkinson Disease: A Randomized Controlled Trial. Including Invited Commentary with Author Response. | Duplicate |
| Zanardi da Silva, 2016 | Parkinson's disease: effects of Aquatic Exercise Dual Task in balance and functionality. | Trial registration |
| Zanardi da Silva, 2020 | Effects of a dual-task aquatic and terrestrial physical exercise program on motor and cognitive functions of individuals with Parkinson's Disease - AquaDualPark study. | Trial registration |
| Özden, 2021 | Comparison of virtual reality rehabilitation and conventional rehabilitation in Parkinson's disease: a randomised controlled trial | Letter to the Editor |
