# Supplementary material for: Effects of motor–cognitive training on dual-task performance in people with Parkinson’s disease: a systematic review and meta-analysis
Source: J Neurol. 2023 Feb 23;270(6):2890–907. doi: 10.1007/s00415-023-11610-8 (PMC10188503; doi:10.1007/s00415-023-11610-8)
Supplement: Supplementary file 4 — Supplementary file4 (DOCX 18 KB) [file 415_2023_11610_MOESM4_ESM.docx]

## Online Resource 4.

**Table 2**. Sensitivity analyses, fixed versus random effects model.

| **Dual task outcome** | **Number of included studies** | **Fixed effects model,**  **MD (95% CI); p; I^2^** | **Random effects model,**  **MD (95% CI); p; I^2^** |
| --- | --- | --- | --- |
| Gait speed (m/s) | 8 | 0.12 (0.08, 0.16); <0.01; 12% | 0.12 (0.08, 0.17); <0.01; 12% |
| Cadence (steps/min) | 5 | 2.91 (0.08, 5.73); 0.04; 0% | |
| Stride length (cm) | 6 | 8.89 (5.37, 12.41); <0.01; 49% | 10.12 (4.86, 15.38); <0.01; 49% |
| Stride length SD | 2 | -0.38 (-1.29, 0.53); 0.42; 0% | |
| Stride time SD | 2 | -0.01 (-0.02, 0.01); 0.26; 0% | |
| Double support (%) | 3 | -1.19 (-2.44, 0.05); 0.06; 48% | -1.63 (-3.76, 0.49); 0.13; 48% |
| Cost on gait speed (%) | 2 | -8.75 (-14.57, -2.92); <0.01; 0% | |
| TUG cog (sec) | 3 | -2.16 (-4.05, -0.27); 0.02; 31% | -1.81 (-4.38, 0.75); 0.17; 31% |
| Reaction time (ms) | 2 | 24.91 (-43.02, 92.84); 0.47; 0% | |
| Cost on stride length (%) | 1 | -6.70 (-12.91, -0.49); 0.03; NA | |
| Accuracy (%) | 1 | 0.90 (-1.96, 3.76); 0.54; NA | |
| Cost on accuracy (%) | 1 | 2.13 (-6.39, 10.65); 0.62; NA | |
| Unipedal stance test (sec) | 1 | 5.70 (-8.19, 19.59); 0.42; NA | |

Abbreviations: NA – Not applicable; TUG cog – Timed Up and Go cognitive

**Table 3**. Sensitivity analysis, with and without passive control group, using random effects model.

| **Dual task outcome** | **Number of included studies, with/without passive control group** | **With passive control group,**  **MD (95% CI); p; I^2^** | **Without passive control group,**  **MD (95% CI); p; I^2^** |
| --- | --- | --- | --- |
| Gait speed (m/s) | 8/7 | 0.12 [0.08, 0.17]; <0.01; 12% | 0.13 [0.07, 0.18]; <0.01; 24% |
| Cadence (steps/min) | 5/4 | 2.91 [0.08, 5.73]; 0.04; 0% | 2.75 [-0.26, 5.77]; 0.07; 0% |
| Stride length (cm) | 6/5 | 10.12 [4.86, 15.38]; <0.01; 49% | 10.61 [3.95, 17.26]; <0.01; 59% |
| Stride length SD | 2/1 | -0.38 [-1.29, 0.53]; 0.42; 0% | -0.28 [-1.58, 1.02]; 0.67; NA |
| Stride time SD | 2/1 | -0.01 [-0.02, 0.01]; 0.26; 0% | -0.01 [-0.03, 0.01]; 0.23; NA |
| Double support (%) | 3/2 | -1.63 [-3.76, 0.49]; 0.13; 48% | -2.97 [-9.45, 3.52]; 0.37; 67% |
| TUG cog (sec) | 3/1 | -1.81 [-4.38, 0.75]; 0.17; 31% | -2.36 (-6.85, 2.14); 0.30; NA |

Abbreviations: NA – Not applicable; TUG cog – Timed Up and Go cognitive
